# LIM domain-containing 2 (LIMD2) promotes the progress of ovarian cancer via the focal adhesion signaling pathway

**DOI:** 10.1080/21655979.2021.2000732

**Published:** 2021-12-10

**Authors:** Lixin Chen, Ji Qian, Qinghua You, Jie Ma

**Affiliations:** a School of Pharmaceutical Sciences, Jilin University, Changchun, Jilin, China; b Bio-teq Center, Fudan University, Shanghai, China; c Department of Pathology, Shanghai Pudong Hospital, Shanghai, China

**Keywords:** Ovarian cancer, LIMD2, progress, focal adhesion

## Abstract

Ovarian cancer (OC) is the leading cause of death from gynecological cancer. In this study, we aimed to explore the role and potential mechanism of LIMD2 during the progression of OC. The expression of LIMD2 was analyzed by GEPIA (Gene Expression Profiling Interactive Analysis) database. Western blot and real-time PCR were applied to detect the gene expression of *LIMD2* in OC cell lines. Cell counting kit-8 (CCK-8) assay, transwell, wound healing assays, and tumor xenograft experiments were used to evaluate the function of LIMD2 in *vitro* and *vivo*. Further, the LIMD2-associated pathways in OC were predicted by RNA-seq analysis, and the involvement of the corresponding cell signaling activities were confirmed by Western blot. We found that LIMD2 was high expressed in OC. Additionally, we found that silencing of LIMD2 inhibited OC cell proliferation *in vitro* and reduced the growth of its xenograft tumors. Moreover, knockdown of LIMD2 significantly decreased the migration of OC cells. Kyoto Encyclopedia of Genes and Genomes (KEGG) pathway analysis revealed that pathways regulating extracellular matrix (ECM)-receptor interactions and focal adhesion signaling, were deregulated by LIMD2. Particularly, we confirmed that reducing LIMD2 could decrease the expression of Focal adhesion kinase (FAK) pathway related molecules. In conclusion, LIMD2 promotes the proliferation and invasion of ovarian cancer *in vitro* and *in vivo*, potentially through regulating the focal adhesion signaling pathway.

## 1 Introduction

Ovarian cancer is the most lethal gynecologic cancer, reported with 313,959 newly diagnosed cases worldwide in 2020, leading to 207,252 deaths [1]. There are little or very mild symptoms at the early stage of OC, making it difficult to be noticed or medically detected [2]. In most cases, the disease has spread beyond the primary site at the time of diagnosis, with metastatic lesions in the pelvic and abdominal cavities [3,4]. Multiple approaches have been applied to the treatment of OC, including surgery, chemotherapy, radiotherapy and targeted therapy. However, metastasis and related complications lead to a 5-year survival rate of only 47% for OC patients [5,6]. Therefore, the development of effective therapeutic targets are imperative for the management of OC.

LIM domain-containing 2 (LIMD2) is an important member of the LIM domain-containing protein family [7], which was shown to be located in the cell membrane and nucleus [8]. LIM domain proteins play important roles in a variety of fundamental biological processes, including cytoskeleton organization, cell lineage specification, cell adhesion, cell motility, and they can also translocate to the nucleus to regulate transcription [9]. In addition, LIMD2 plays an important role in tumorigenesis and development. It has been reported that LIMD2 was a lymph node metastasis marker for papillary thyroid carcinoma (PTC) [10]. Also, LIMD2 was found to be overexpressed in most metastatic lymph nodes in PTC, while it was undetectable or expressed at relatively low levels in the primary PTCs [7]. In addition, LIMD2 has been characterized as an important cancer progression regulator in melanoma, bladder cancer, non-small cell lung cancer and breast cancer [11–13]. Nevertheless, the functions and potential molecular mechanisms of LIMD2 in OC are still not clear.

In the present study, we aimed to investigate the role of LIMD2 in ovarian cancer. First, we examined the expression of LIMD2 by GEPIA database, western blot and RT-PCR. Next, we investigated the function of LIMD2 *in vitro* and *in vivo*. Finally, by RNA-seq, Western blot and inhibitor assay, we explored the regulatory signaling pathway of LIMD2. These findings may help for understanding the progression of OC and shed some light on the targeted therapy against this deadly malignancy.

## 2 Materials and methods

### 2.1 Cell culture

The OC cell lines A2780, HO8910, HO8910PM, SK-OV-3 and OVCAR-3 were obtained from ATCC and maintained in RPMI 1640 medium (Thermo Fisher Scientific, USA) supplemented with 10% fetal bovine serum (FBS, Thermo Fisher Scientific, USA) and glutamine (Thermo Fisher Scientific, USA) or in Dulbecco’s modified Eagle’s medium (DMEM, Thermo Fisher Scientific, USA) supplemented with 10% FBS and antibiotics (Thermo Fisher Scientific, USA). Normoxic cell incubation (Thermo Fisher Scientific, USA) was performed at 37°C in 5% CO_2_ with 95% humidity.

### 2.2 Immunofluorescence

The five OC cell lines expressing LIMD2 were processed for immunofluorescence based on similar conditions to the ones described above [14]. The cultured cells were fixed with 4% paraformaldehyde for 10 min at room temperature. Subsequently, the cells were permeabilized with 0.01% Triton X-100 and blocked with 3% BSA for 1 h at room temperature. The primary antibody against LIMD2 (catalog number: 15,471-1-AP; Proteintech, China) was incubated overnight at 4°C and Alexa Fluor 555-labeled secondary antibody (A-21428, Thermo Fisher Scientific, USA) was incubated for 1 h at room temperature. Slides were mounted with DAPI (Sigma-Aldrich, Germany) and visualized using an Olympus microscope (Olympus, Japan).

### 2.3 Knockdown of LIMD2 expression with shRNA

LIMD2 expression in A2780 and HO8910PM cells was knocked down by transfection with lentivirus-packaged shRNA. Five LIMD2-targeting shRNAs and a control shRNA were designed and packaged with the pLVX-shRNA2 lentivirus system. The five shRNAs specifically targeting LIMD2 are: shLIMD2-1,5ʹ-AAGCAGCACAAGGAGCTCTGG-3ʹ; shLIMD2-2, 5ʹ-AAGCACTGTCACACCAAGCTC-3ʹ shLIMD2-3, 5ʹ-AAGAGCAAAGGCAACTACGAC-3ʹ; shLIMD2-4, 5ʹ-GGTCTCAGATGGCAAGGATCA-3ʹ; shLIMD2-5, 5ʹ-AACTCTTGCTTCTGCTGCAAG-3ʹ.The DNA oligonucleotides of target sequence was synthesized by Shanghai GeneRay Biotech Co., Ltd. Cells were transfected with shRNA lentiviruses for 24 h followed by fresh medium replacement. The full length of LIMD2 was cloned into the psLenti-EF1a-EGFP-F2A-puro-CMV-MCS, and the lentivirus was produced as previously described [15]. The lentivirus exhibiting LIMD2 overexpression was transfected into A2780 and SKOV3 cells, and the positively transduced cells were selected using puromycin. Seventy-two hours after transfection, cells stably transfected with lentivirus were selected with puromycin (5 µg/ml) for 24 h. Then, the cells were cultured for another 48 h, and the expression of LIMD2 was evaluated by real-time PCR at the mRNA level and western blot at the protein level.

### 2.4 Real-time PCR (RT-PCR)

Cultured cells were washed once with ice-cold PBS and collected with TRIzol reagent (Thermo Fisher Scientific, USA). Total RNA of cells was isolated with chloroform. The quantity and concentration of RNA were evaluated with a NanoDrop system (Thermo Fisher Scientific, USA). A total of 1 μg RNA was reverse transcribed into cDNA with PrimeScript™ RT Master Mix (TaKaRa, Japan) [16]. The mRNA levels of the genes were quantified with a real-time PCR kit (Tiangen, China) on a Bio-Rad CFX96 real-time PCR system according to the manufacturer’s instructions. GAPDH was used as the internal control. The sequences of specific primers used in this study are listed below: LIMD2-F: 5′-CAGGAAGACCCTACCAAATATC-3ʹ LIMD2-R: 5ʹ- CCCAACAGGGCTGATTTAC-3ʹ GAPDH-F: 5ʹ-GTATGACAACAGCCTCAAGAT-3ʹ GAPDH-R: 5ʹ-GTCCTTCCACGATACCAAAG-3ʹ. Three biological replicates were performed.

### 2.5 Cell proliferation assay [17]

Cell proliferation was evaluated by the CCK-8 (Targetmol, USA) assay. The A2780 cells (Control and shLIMD2) were passaged to 96-well plates (1 × 10^3^ cells/well) and cultured for the indicated time periods (24 h, 48 h, 72 h, 96 h, 120 h) before the addition of CCK-8 (5 mg/ml, 10 μL/wells) reagent. The absorbance of all the wells at 450 nm was detected with a Thermomax microplate reader (Molecular Devices, Sunnyvale, USA). Three biological replicates were performed.

### 2.6 Wound healing assay [18]

The cells (1.0 × 10^6^/well) were passaged into 6-well plates and cultured to a confluent monolayer. The cell (shLIMD2 and control) monolayer was scraped in a straight line with a pipette tip, the debris was washed and removed with PBS (Sangon biotech, China) as previous description [19]. Then, the cells were cultured with serum-free medium and imaged at 0 and 48 h. The migration rate of the cells was measured with the ImageJ software according to the manufacturer’s instructions (NIH, USA). Three biological replicates were performed.

### 2.7 Transwell migration assay [20]

The cells (4 × 10^3^/well, Control and shLIMD2) in 200 μL of serum-free medium were added to the top chambers of the transwell (8-μm pore size; Corning, USA). 500 μL of DMEM supplemented with 20% FBS was added to the lower chambers to induce cell migration. Then, the cells were cultured for another 48 h, the cells that migrated through the transwell filter were fixed with 4% PFA and stained with a 0.1% crystal violet solution. The images of the migrated cells were captured with the microscope. Three biological replicates were performed.

### 2.8 Western blot analysis [21]

Cell were lysed with RIPA lysis buffer (C500005, Sangon biotech, China). Total protein (20 μg) was separated on a sodium dodecyl sulfate (SDS)-polyacrylamide gel and transferred onto a 0.45 μm PVDF membrane (Merck, Germany). The membranes were blocked in 5% nonfat milk solution for 1 h at room temperature and were then incubated with the primary antibody at a 1:1000 dilution overnight at 4°C. Then the goat anti-Rabbit HRP-labeled secondary antibody (#4030-05, SouthernBiotech, Birmingham, USA) was incubated for 2 h at room temperature. The ECL regents (Pierce, Rockford, IL, USA) was used to detect the signal of the target proteins. The specific primary antibodies were purchased from the following resource: anti-LIMD2 (#ab167895, Abcam, Cambridge, UK), anti-FAK(#12,636-1-AP,proteintech, HuBei, China), anti-Phospho-FAK(Tyr397)(#8556 T,CST, Boston, USA), anti-B-tubblin(HRP-66031, Proteintech, HuBei, China), anti-RAC1(#24,072-1-AP,Proteintech, HuBei, China) and Tensin 2 Antibody (#11,990, CST,Boston, USA).

### 2.9 Tumor xenograft experiments

The animal experiments were followed the Guide for the Care and Use of Laboratory Animals published by the National Institutes of Health (NIH, USA) and approved by the Ethics Committee of Experimental Research at Jilin University. 6-week-old female nude mice (six mice/group, grouped as control and shLIMD2) were injected subcutaneously in the bilateral flank area with 5 × 10^6^ cells in 200 μL of normal saline. Tumor growth was monitored every 3 days, and the tumor volume was calculated. After injection for six weeks, the mice were euthanized and the weight of tumor was measured. Each tumor was dissected for subsequent histological examination and molecular studies as previously description [19]. For the *in vivo* tumor metastasis assay, the nude mice per group (n = 6) were injected intraperitoneally (*i.p*.) with 5 × 10^6^ cells in 200 μL of normal saline as previous description [22,23]. Seven weeks after the *i.p*. injection, the visceral organs (liver, intestine, mesentery, kidney, ovary and diaphragm) were observed. The visceral organs were removed for subsequent histological examination.

### 2.10 Immunohistochemical Staining [24]

Paraffin-embedded tissue was sliced and dewaxed. After antigen retrieval, primary antibodies were incubated with slides at 4°C overnight. Herein, anti-LIMD2 (#ab167895, Abcam, Cambridge, UK) were used. After washing, secondary antibodies were incubated at 37°C for 30 min and washed. Then diaminobenzidine (DAB) was applied for color development. Lastly, all sections were scanned by Pannoramic DESK, P-MIDI, P250, P1000 (3D HISTECH; Hungary) and were read by Pannoramic Scanner (3D HISTECH; Hungary).

### 2.11 Statistical analysis

In this study, the unpaired t-test was used to evaluate the significance of the differences between two groups, while ANOVA was used to evaluate the significance of the differences between three groups with the Prism software (GraphPad 8.0 Software, USA). A p value of 0.05 or less is considered significant. The thresholds of |log2-fold fold change |>1.5 and P-adjusted < 0.05 were used to identify significantly differentially expressed genes (DEGs).

## 3 Results

In this study, we aimed to investigate the role of LIMD2 in OC. We found LIMD2 was upregulated in OC tissue compared with normal ovary tissues. Knockdown of LIMD2 inhibited OC cell proliferation and migration. Additionally, knockdown of LIMD2 could suppresses tumor through focal adhesion pathway in OC.

### 3.1 The expression of LIMD2 in OC tissues and cell lines

**Figure 1.**
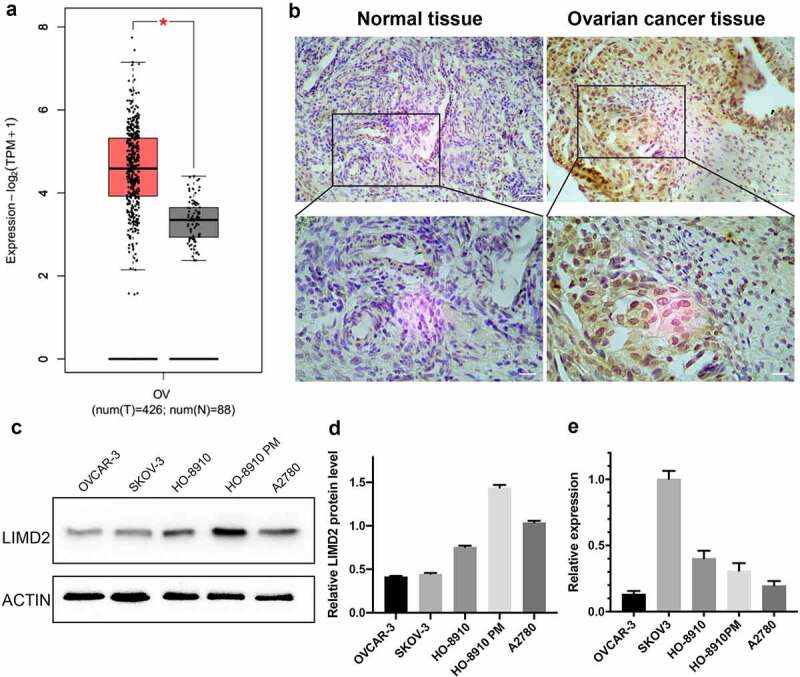
**Expression of LIMD2 in ovarian cancer tissues and cell lines**. (a) The expression of LIMD2 in ovarian cancer tissues was significantly higher than that in normal ovarian samples from the TCGA by using GEPIA database. (b) The immumohistochemical staining of LIMD2 in ovarian cancer tissue and normal control. Bar = 100 μm. (c) Analysis of the mRNA expression of LIMD2 in ovarian cancer cell lines by real-time PCR. (d-e) Analysis of the expression of LIMD2 in ovarian cancer cell lines by Western blot. *P < 0.05, **P < 0.01 versus the control group

By GEPIA database, we found that the expression of LIMD2 was significantly increased in OC compared with normal ovary tissues (P < 0.05) (Figure 1a). In addition, the immumohistochemical staining showed that LIMD2 was highly expressed in OC tissues (Figure 1b). Otherwise, in oder to validate the expression of LIMD2 in ovarian cancer lines, five OC cell lines were selected. LIMD2 mRNA was highly expressed in SKOV3 cells, followed by HO8910, HO8910PM, A2780 and OVCAR-3 cells (Figure 1c). At the protein level, LIMD2 was widely expressed in all five OC cell lines, while its levels in A2780 and HO8910PM cells were higher than other three cell lines (Figures 1D **and 1E**). Thus, LIMD2 is commonly expressed both in OC tissues and OC cell lines.

### 3.2 The distribution of LIMD2 in OC cells

**Figure 2.**
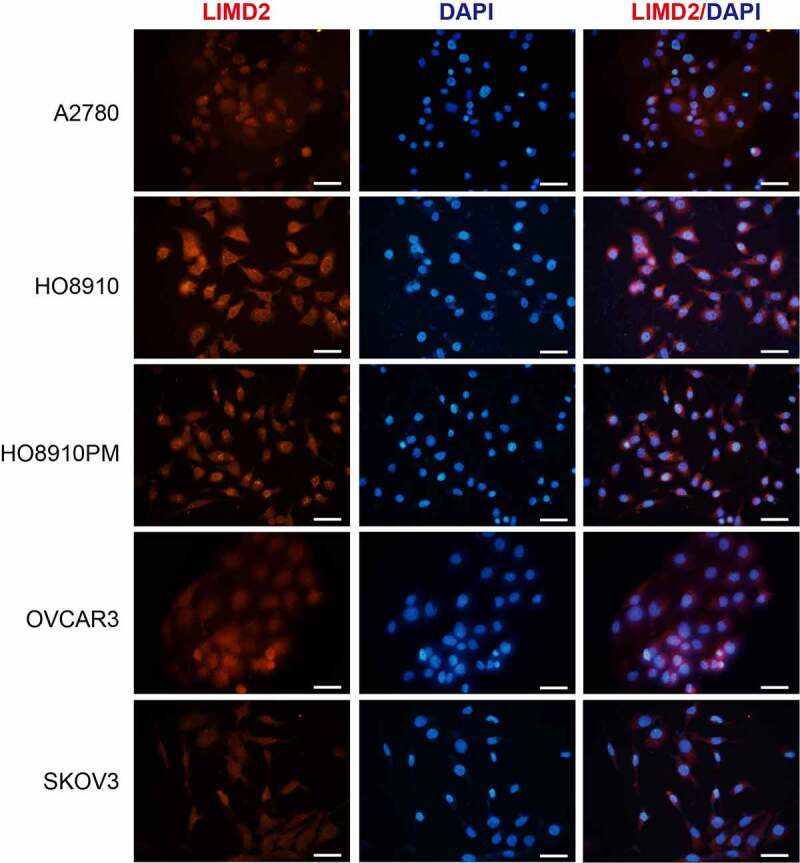
**The expression and distribution of LIMD2 in ovarian cancer cell lines**. The LIMD2 protein in ovarian cancer cells was analyzed by immunofluorescence. Bar = 50 μm

Next, we investigated the subcellular distribution of LIMD2 in OC cells. Immunofluorescence was performed in the five OC cell lines to determine the localization of LIMD2 protein (Figure 2). We found that LIMD2 protein was localized on the cell membrane as well as in the cytoplasm of all the OC cell lines, while it was barely visible in the nucleus (Figure 2). These finding indicated that LIMD2 exhibited unique expression profile in OC cells, relative to the previous reported cancer types.

### 3.3 Knockdown of LIMD2 inhibits OC cell proliferation and migration in vitro

**Figure 3.**
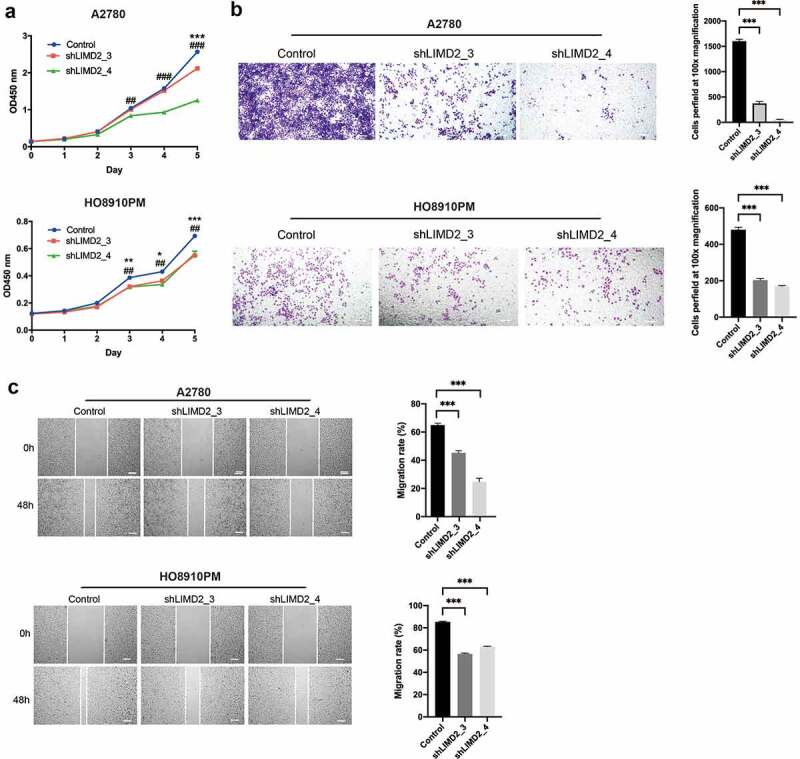
**Knockdown of LIMD2 inhibits the proliferation and migration of ovarian cancer cells *in vitro.*** (a) Knockdown of LIMD2 inhibits A2780 and HO8910PM cells growth by CCK-8 assay. (b) Transwell assays revealed that the invasion capacity of A2780 and HO8910PM cells transfected with shRNA-LIMD2 was lower than that of control cells. (c) Wound healing assay indicated that shRNA-LIMD2 significantly inhabited cell migration in A2780 and HO8910PM cells compared with that in control cells. **P < 0.01, ***p < 0.001. Bar = 100 μm

To investigate whether LIMD2 regulate OC progression, knockdown and overexpression tests were performed on OC tumor cells. We firstly knocked down LIMD2 in A2780 and HO8910PM cells. By the CCK-8 assay, we found that knockdown of LIMD2 inhibits the proliferation of the two OC cell lines (P < 0.05) (Figure 3a). Otherwise, overexpression of LIMD2 promoted A2780 and SKOV3 cell proliferation (**Fig. S1A**). Next, transwell migration assays were performed to determine effect of LIMD2 on the migration. As shown in Figure 3b, the migrated cells were significantly reduced with the knockdown of LIMD2. In addition, overexpression of LIMD2 promoted A2780 and SKOV3 migration (**Fig. S1B**). Furthermore, we performed the wound healing assays on the OC cells with control and shLIMD2 and found that the migration ability was significantly decreased upon LIMD2 knockdown (P < 0.05) (Figure 3c). However, overexpression of LIMD2 yielded the opposite result. These data suggested that LIMD2 was a functional regulator of OC cells proliferation and migration.

### 3.4 Knockdown of LIMD2 inhibits the tumorigenic and metastatic potential in vivo

**Figure 4.**
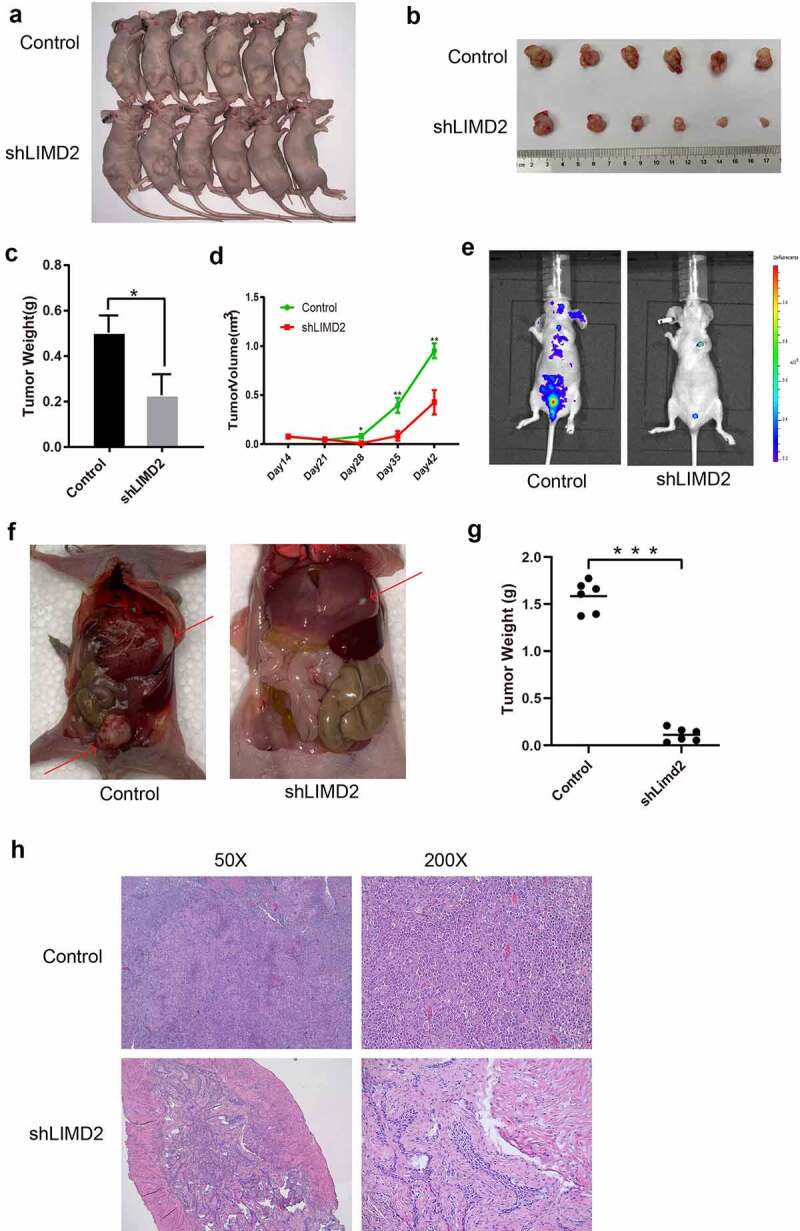
**Knockdown of LIMD2 inhibits the growth and metastasis of ovarian cancer cells *in vivo***. (a-d) Morphology, volume change and weight of mouse xenograft tumors derived from subcutaneous transplantation of shLIMD2 and control A2780 cells for 42 days. (e-g) Morphology and weight of the mouse xenograft model of ovarian cancer metastasis derived by intraperitoneal injection of shLIMD2 and control A2780 cells. Significant metastasis progression within the groups was also monitored using the intensity of light emission from the luciferase enzyme expression of tumor cells via IVIS in (e). The larger the red area, the more invasive the tumor cells. The red arrow in (f) indicates the metastatic tumor. The control group has more and larger tumors than that in shLIMD2 group. ***p < 0.001. (h) The HE staining of tumor and metastatic lesions of nude mice

Consistent with the above *in vitro* findings, we proceeded to test the effect of LIMD2 knockdown on the tumorigenesis and metastasis of OC cells. The LIMD2-knockdown (shLIMD2_4) or control A2780 cells were subcutaneously injected into athymic nude mice. We found that LIMD2 silencing was associated with significant reduced levels of tumor size and tumor weight (P < 0.05) (Figures 4A**-4D**). Additionally, a metastatic model with intraperitoneal injection of A2780 cells was examined. We found that the control cells established visible metastatic tumors at multiple loci (Figures 4E **and 4 F**). However, A2780 cells with LIMD2 knockdown generated fewer metastatic lesions, which was supported by the collective tumor weight shown in Figures 4G **and 4 H**. Overall, knockdown of LIMD2 in A2780 OC cell line inhibited its tumorigenesis and metastasis *in vivo*.

### 3.5 Knockdown of LIMD2 suppresses tumor through focal adhesion pathway

**Figure 5.**
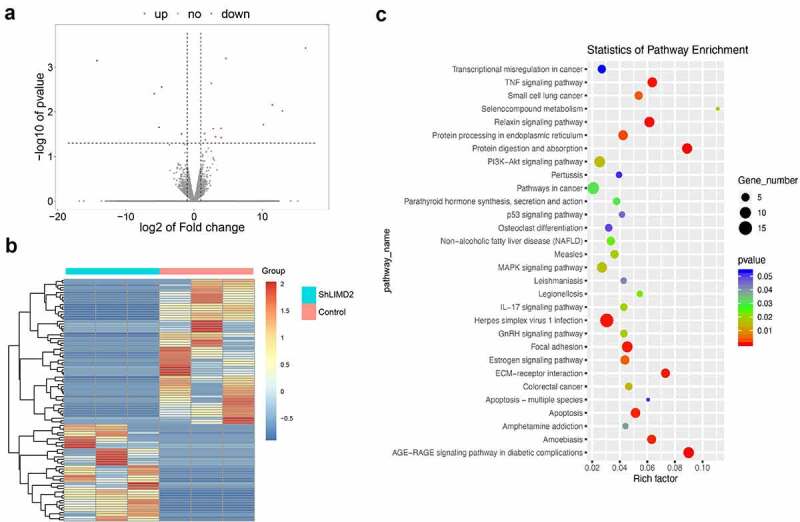
**The differential genes and KEGG analysis in LIMD2 knockdown cells**. (a) The volcano plot of the genes between hLIMD2 and control A2780 cells. (b) The heatmap of the differentially expressed genes between hLIMD2 and control A2780 cells. (c) KEGG pathway analysis of the differentially expressed genes between shLIMD2 and control A2780 cells

**Figure 6.**
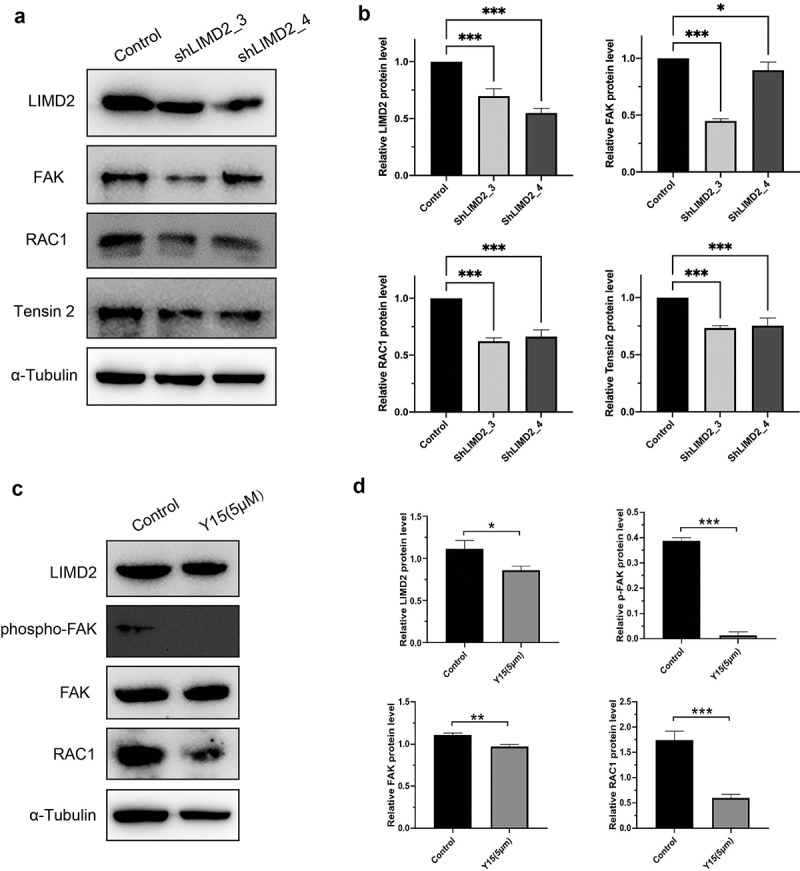
**The regulation of focal adhesion pathway in LIMD2 knockdown cells**. (a) Analysis of the expression of focal adhesion signaling proteins in shLIMD2 and control A2780 cells by Western blot. (c) The protein analysis of focal adhesion signaling pathway affect by specific inhibitor Y15

To explore the potential mechanism of LIMD2 in OC, RNA- seq was performed to find the changes between LIMD2 shRNAs or control vectors in A2780 cells. The volcano plot and heatmap showed the differentially expressed genes between hLIMD2 and control A2780 cells (Figures 5A **and 5B**). KEGG analysis showed that signaling of the extracellular matrix (ECM)-receptor interaction and focal adhesion were both greatly impacted by silencing LIMD2 (Figure 5c). Furthermore, we evaluated the Focal adhesion signaling pathway activity by Western blot. The results showed that silencing LIMD2 in A2780 cells led to markedly reduced expression of FAK, RAC1 and Tensin 2 (Figures 6A **and 6B)**. What’s more, after treating the cells with the FAK pathway specific inhibitor Y15, we found that the expression of LIMD2 decreased as well as p-FAK and RAC1 (Figures 6C **and 6D)**. These may suggest that LIMD2 promote OC cell proliferation and metastasis through the focal adhesion pathway.

## 4 Discussion

The high potential of recurrence and the low survival rates of OC patients in advanced stages have made OC the third deadliest gynecological cancer, with five-year survival rates below 45% [25,26]. Novel markers or genes involved in OC metastasis and growth have been shown to help the malignancy grading and to facilitate the development of precision medicine for OC [27–30]. In this study, we investigated the potential function and regulatory mechanism in OC.

As a LIM domain protein, LIMD2 has been functionally associated with cell motility and adhesion, mostly by regulating mRNA transcription [9,31]. In our study, we found that LIMD2 was upregulated in OC tissues, which was consistent with the findings in lung cancer [11] and papillary thyroid cancer [10]. LIMD2 expression levels were positively correlated with cell motility metastatic potential and grade in both fresh and archived tumors, including bladder melanoma breast and thyroid tumors [13]. Otherwise, several studies have proposed that LIMD2 correlates with the malignant progression of various types of cancer. LIMD2 regulates key steps of metastasis cascade in papillary thyroid cancer cells via MAPK crosstalk [32]. Also, LIMD2 promotes the proliferation and invasion of non-small cell lung cancer cells [11,12] . In our study, by using CCK-8 assay, transwell, wound healing assays and tumor xenograft experiments, we found that LIMD2 could promote proliferation and metastasis of ovarian cancer in vitro and vivo. This was similar to what has been found in other cancers.

Subsequently, to understand the underlying mechanism of LIMD2 in regulating the progress of OC, RNA-seq was performed. By KEGG analysis, we found that the focal adhesion pathway may play an important role. Focal adhesion kinase (FAK) is a non-receptor tyrosine kinase that resides at the sites of integrin clustering, known as focal adhesions [33]. The activation of FAK are usually investigated in primary or metastatic cancers and correlated with the poor clinical outcome [34]. It has been reported that FAK promotes OC tumor initiation and invasion [35–37]. In addition, FAK is involved in protecting OC cells from anoikis and apoptosis, which contribute to OC progression in patients [38,39]. What’s more, FAK interacts with the ECM of the basement membrane, which contains fibronectin for the docking of metastastic OC cells [39–42]. In our study, we found FAK pathway related proteins were higher than that in LIMD2 knockdown group. Our results indicated an association of LIMD2 and FAK signal pathway, which may suggest a potential molecular link between LIMD2 and the metastatic ability of OC.

## 5. Conclusion

In conclusion, we demonstrated that knockdown of LIMD2 inhibited cell growth and metastasis in OC. And it may play a role by downregulating the focal adhesion pathway. However, there are still some shortcomings of this work. For example, the target of LIMD2 needs to investigate. In addition, more clinical samples should be validate to explore the prognostic value of LIMD2 in OC.

## Supplementary Material

Supplemental Material

## Data Availability

The datasets analyzed during the current study are available from the corresponding author on reasonable request.The RNA-seq was uploaded to NCBI database. The URL: https://www.ncbi.nlm.nih.gov/geo/query/acc.cgi?acc=GSE176462.

## Highlights

- LIMD2 was upregulated in ovarian cancer.
- LIMD2 promotes the proliferation and migration of ovarian cancer *in vitro* and *in vivo*.
- Focal adhesion signaling pathway was regulated by LIMD2 in ovarian cancer.
