# Supplementary figures and images for: LIM domain-containing 2 (LIMD2) promotes the progress of ovarian cancer via the focal adhesion signaling pathway
Source: Bioengineered. 2021 Dec 10;12(2):10089–100. doi: 10.1080/21655979.2021.2000732 (PMC8809939; doi:10.1080/21655979.2021.2000732)

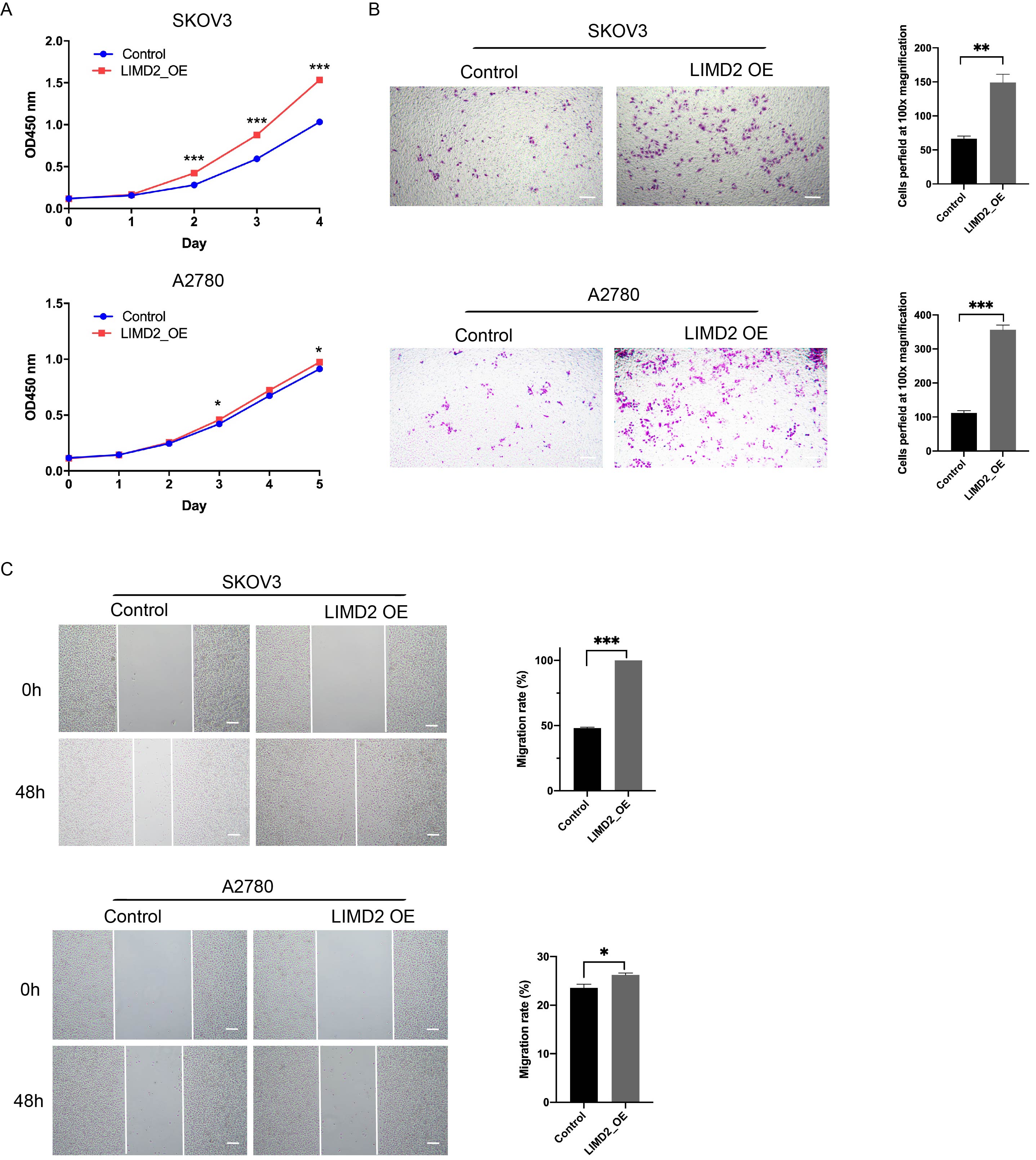

Supplement: Supplemental Material [file KBIE_A_2000732_SM9143.jpg]
